# FOXH1 Is Regulated by NANOG and LIN28 for Early-stage Reprogramming

**DOI:** 10.1038/s41598-019-52861-8

**Published:** 2019-11-11

**Authors:** Ling Wang, Yue Su, Chang Huang, Yexuan Yin, Jiaqi Zhu, Alec Knupp, Alexander Chu, Young Tang

**Affiliations:** Department of Animal Science, Institute for Systems Genomics, University of Connecticut, 1390 Storrs Rd, Storrs, CT 06269 USA

**Keywords:** Induced pluripotent stem cells, Reprogramming

## Abstract

FOXH1 is a primitive-streak specifier and ACTIVIN co-effector that plays an important role in development, and positively regulates the generation of human induced pluripotent stem cells (iPSCs) from somatic cells by OCT4, SOX2, KLF4, and MYC (OSKM) transduction. However, the mechanism and upstream regulation for FOXH1 expression in reprogramming are unclear. We found FOXH1 expression plays a significant role to enhance epithelial marker and suppress mesenchymal gene expression in OSKM-mediated human cell reprogramming. Furthermore, NANOG and LIN28 (NL) co-stimulate FOXH1 expression, which correlates with the enhanced reprogramming efficiency by NL-factors. FOXH1 expression is also stimulated by a specific inhibitor for H3K79 methyltransferase DOT1L (iDOT1L) but not by inhibition of the canonical WNT signaling. We further show that blocking endogenous FOXH1 expression eliminates the enhanced reprogramming effect by NL and iDOT1L. However, overexpressing FOXH1 in NL plus iDOT1L condition results in significantly reduced TRA-1-60 positively expressed cells and decreases pluripotent marker expression in reprogramming. Our study elucidated an essential role for properly stimulated FOXH1 expression by NANOG, LIN28, and H3K79 demethylation for dramatic enhancement of reprograming.

## Introduction

Two gene cocktails including OCT4, SOX2, KLF4, c-MYC (OSKM)^1,2^ and OCT4, SOX2, NANOG, LIN28A (OSNL)^3^ can reprogram somatic cells to embryonic stem cell (ESC)-like, induced pluripotent stem cells (iPSCs). FOXH1 is an important binding-partner of SMAD2 that mediates ACTIVIN/NODAL signaling for anterior primitive-streak development in mouse embryos^4,5^, and plays a key role in forebrain patterning and retinoic acid signaling in mice^6^. Interestingly, the level of endogenous FOXH1 stimulation is proportional to OSKM-mediated reprogramming efficiency^7^. Mechanism-wise, FOXH1 was shown to downregulate fibroblast marker CD13 and stimulate epithelial marker EPCAM expression in reprogramming^7^. However, little is known about the upstream regulators of FOXH1 during the reprogramming as well as in development processes. Also, whether FOXH1 regulates mesenchymal to epithelial transition (MET)^8–10^, an important event for iPSC generation is not clear.

In this study, we used primary human mesenchymal stem cells (MSCs) that exhibited low reprogramming efficiency when transduced by retroviral OSKM. We used Alkaline Phosphatase (AP)-staining, and also fluorescent staining of TRA-1-60, the glycoprotein expressed exclusively in human iPSCs/ESCs and one of the most reliable markers for primed-state pluripotency^11,12^ and successful reprogramming^13,14^ to monitor the reprogramming process. We found FOXH1 significantly stimulates MET in reprogramming. Also, NANOG and LIN28 (NL) co-stimulate the expression of FOXH1 for human iPSC generation. This effect can be reinforced by inhibition of histone H3K79 methyltransferase DOT1L. We further found endogenous FOXH1 expression is necessary for the enhanced reprogramming effect of NL and inhibition of DOT1L, but overexpressing FOXH1 in NL and iDOT1L condition greatly reduces TRA-1-60 positive (+) cell population in reprogramming. Our study elucidated the importance of appropriate level of FOXH1 expression for NL and H3K79 demethylation-enhanced reprogramming.

## Results

### FOXH1 stimulates MET in OSKM-mediated reprogramming

**Figure 1:**
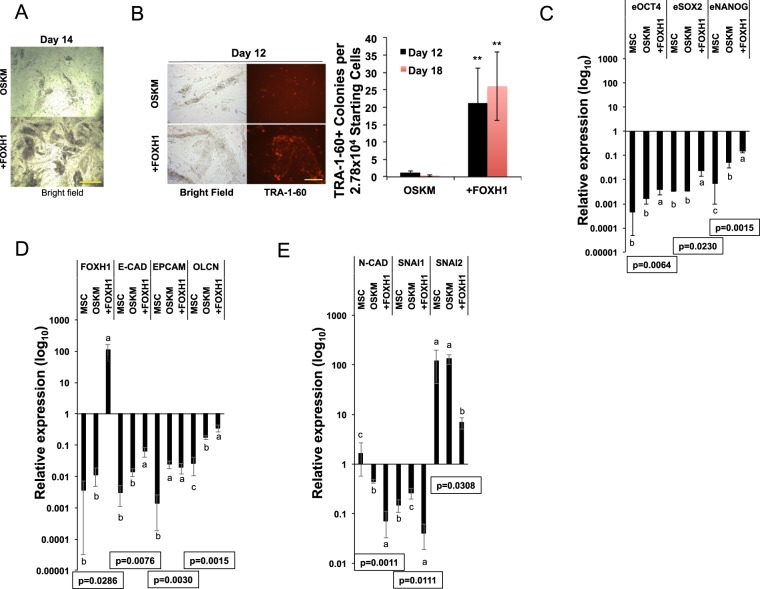
Effects of FOXH1 on OSKM-mediated Reprogramming. (**A**) Representative images of colony formation in OSKM and OSKM + FOXH1 (shown as +FOXH1) conditions on reprogramming day 14. Scale bar: 625 μm. (**B**) Left: Images of TRA-1-60 immunostaining on OSKM and +FOXH1 induced colonies on reprogramming day 12. Scale bar: 250 μm. Right: Numbers of TRA-1-60 positive colonies in OSKM and +FOXH1 reprogramming conditions on day 12. Bars represent mean ± s.d., n = 3. **p < 0.01. (**C**) qRT-PCR for endogenous (e) core pluripotent gene expression in OSKM- and +FOXH1-mediated reprogramming on day 14. Bars represent mean ± s.d., n = 3. Values are normalized by GAPDH and compared with human H9 ESCs. (**D**) qRT-PCR for endogenous plus transgene FOXH1, and endogenous epithelial gene expression in OSKM- and +FOXH1-mediated reprogramming on day 14. Bars represent mean ± s.d., n = 3. Values are normalized by GAPDH and compared with human H9 ESCs. (**E**) qRT-PCR for mesenchymal gene expression in OSKM-mediated reprogramming. OSKM- and +FOXH1-mediated reprogramming on day 14. Bars represent mean ± s.d., n = 3. Values are normalized by GAPDH and compared with human H9 ESCs.

FOXH1 exerts important roles in regulating the efficiency of OSKM-mediated reprogramming^7^. To understand how FOXH1 expression regulates the reprogramming process, we overexpressed retroviral pMXs-FOXH1 together with OSKM-factors in human MSCs. Ectopic FOXH1 expression caused massive formation of colonies, compared with the OSKM control (Fig. 1A). We counted the TRA-1-60 positive (TRA-1-60+) colony numbers on reprogramming days 12 and 18, and found addition of FOXH1 significantly stimulated the TRA-1-60+ colony formation (Fig. 1B). Quantitative reverse transcription-PCR (qRT-PCR) on reprogramming day 14 cell RNAs showed that the expression of endogenous pluripotent genes OCT4, SOX2, and NANOG was further enhanced by addition of FOXH1 (Fig. 1C). We found that FOXH1 expression significantly stimulated the epithelial markers E-CADHERIN (E-CAD) and OCLN (Fig. 1D), although EPCAM expression was not stimulated by FOXH1 as previously reported in reprogramming human fibroblasts^7^ (Fig. 1D). The reason why we did not detect change in the EPCAM expression could be due to a difference in cell type or time point of reprogramming. Analysis of whole cell population undergoing reprogramming instead of TRA-1-60+ cells could also be the cause of undetected EPCAM upregulation. We further found that FOXH1 expression significantly suppressed the expression of mesenchymal markers N-CAD, SNAI1, and SNAI2 (Fig. 1E). These findings demonstrate that FOXH1 expression promotes MET transition in OSKM-mediated human cell reprogramming. Of note, FOXH1 overexpression reduced the expression of SNAI2, a pre-iPSC marker that prevents complete reprogramming^15^ to ~ 5% of the OSKM condition (Fig. 1E).

### FOXH1 expressions is synergistically stimulated by NANOG, LIN28, and iDOT1L and independent of WNT activity

**Figure 2:**
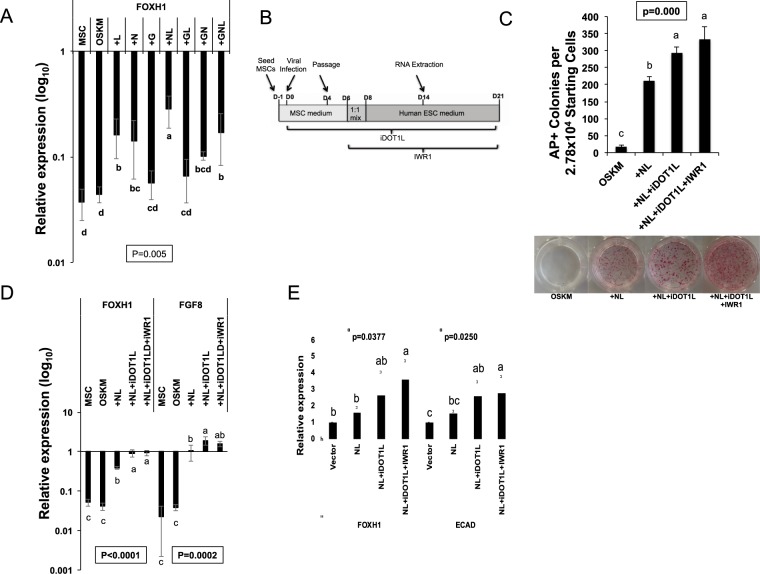
Regulation of FOXH1 expression by NL and iDOT1L in reprogramming. (**A**) qRT-PCR for endogenous FOXH1 expression in OSKM or OSKM plus NANOG, LIN28, GLIS1 or their combinations on reprogramming day 14. Bars represent mean ± s.d., n = 3. Values are normalized by GAPDH and compared with human H9 ESCs. (**B**) Schematic diagram for the timeline of reprogramming human mesenchymal stem cells with iDOT1L (3.3 μM) and IWR1 (2.5 μM) application. (**C**) Top: Numbers of alkaline phosphatase (AP) positive colonies on reprogramming day 21. Bars represent mean ± s.d., n = 3. Bottom: Representative pictures of AP-stain for different reprogramming conditions on day 21. (**D**) qRT-PCR for endogenous FOXH1 and FGF8 expression in different conditions on reprogramming day 14. Bars represent mean ± s.d., n = 3. Values are normalized by GAPDH and compared with human H9 ESCs. (**E**) qRT-PCR for the activation of FOXH1 and ECAD in MSCs by the NL overexpression with or without iDOT1L and IWR1. MSCs were treated for 5 days before harvested for analysis. Values are normalized by GAPDH and compared with control MSCs infected by retrovirus carrying pMXs empty vector.

Additional reprogramming factors such as GLIS1 (G)^16^, NANOG (N)^17,18^, or LIN28 (L)^18^ to OSKM were reported to enhance reprogramming with under-clarified mechanism. We recently showed that among these 3 factors, combined expression of NL most significantly stimulated TRA-1-60+ iPSC colony formation in retroviral OSKM-mediated reprogramming from human MSCs^19^. We wondered whether FOXH1 expression plays a role here for the enhanced reprogramming. We compared FOXH1 gene expression in these reprogrammed cells on day 14. OSKM or addition of G to OSKM (+G) showed little effect on stimulating FOXH1 expression from parental MSCs (Fig. 2A). However, addition of L, N to OSKM (+L, +N) significantly stimulated the expression of FOXH1 (Fig. 2A). Also, addition of NL-factors (+NL) but not GL or GN (+GL or GN) showed add-up effect on FOXH1 expression (Fig. 2A). Thus, NL-factors significantly co-stimulate FOXH1 expression in reprogramming.

Inhibiting chromatin H3K79 methyltransferase DOT1L improves OSKM-induced reprogramming efficiency from human fibroblasts^13^. Also, inhibiting canonical WNT/β-CATENIN signaling at late reprogramming stage prevents differentiation of the reprogrammed cells^20^. We have shown that the DOT1L inhibitor (iDOT1L)^13^ and canonical WNT signaling inhibitor (IWR1)^21^ improve total TRA-1-60+ colony number and the homogeneity of individual TRA-1-60+ colonies, respectively^19^. We wondered if these enhanced reprogramming events involve regulation of FOXH1 expression. iDOT1L and IWR1 were added from initial (day 0) and late-stage (day 6) of OSKM + NL mediated MSC reprogramming, respectively (Fig. 2B). Both treatments significantly improved the alkaline phosphase (AP)-stained colony number (Fig. 2C). qRT-PCR revealed that iDOT1L-treatment significantly stimulated FOXH1 expression compared with the NL condition, which is accompanied with enhanced expression of FOXH1 target gene FGF8^6^ (Fig. 2D). On the other hand, inhibiting WNT activity by IWR1 did not affect FOXH1 expression (Fig. 2D). In addition, to investigate if NL, iDOT1L or the IWR1 can activate FOXH1 or epithelial gene, we overexpressed NL in MSCs with or without application of the chemicals, in the absence of OSKM. We found NL alone or with iDOT1L did not significantly stimulate FOXH1 (Fig. 2E). But strong stimulation for FOXH1 was achieved in the OSKMNL (~7.6 fold over MSCs) and OSKMNL + iDOT1L (~17.8 fold over MSCs) reprogramming conditions (Fig. 2D). Thus, NL and iDOT1L-treatment synergize to upregulate FOXH1 expression independent of WNT activity during reprogramming. The robust activation of FOXH1 by NL and iDOT1L is dependent on the cooperation with OSKM.

### FOXH1 expression is necessary for NL and iDOT1L enhanced human cell reprogramming

**Figure 3:**
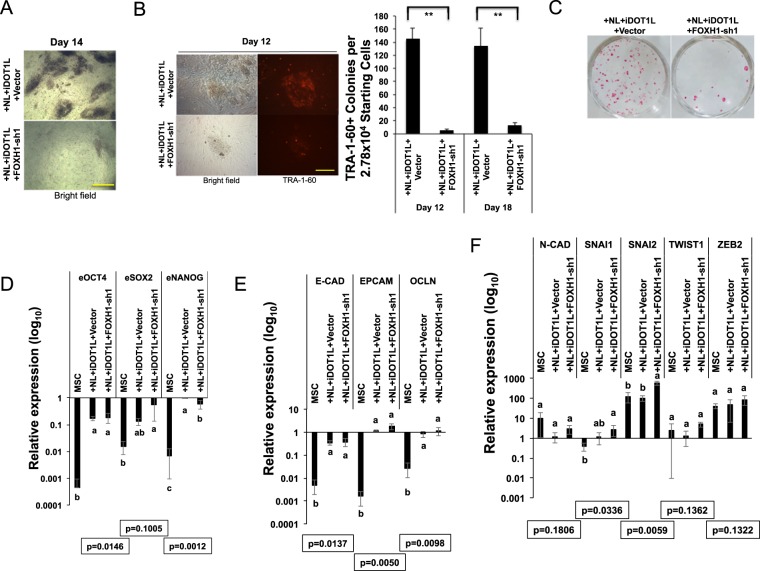
FOXH1 is necessary for NL and iDOT1L enhanced reprogramming. (**A**) Representative images of colony formation in NL + iDOT1L control and FOXH1 depletion conditions on reprogramming day 14. Scale bar: 625 μm. (**B**) Left: Representative images of TRA-1-60 staining on the induced colonies in NL + iDOT1L control and FOXH1 depletion conditions on reprogramming day 12. Scale bar: 250 μm. Right: Numbers of TRA-1-60 positive colonies induced in NL + iDOT1L control and FOXH1 depletion conditions on reprogramming day 12 and 18. Bars represent mean ± s.d., n = 3. **p < 0.01. (**C**) Representative images for AP-stain of induced colonies in NL + iDOT1L control and FOXH1 depletion conditions on reprogramming 18. (**D**) qRT-PCR for endogenous core pluripotent gene expression on reprogramming day 14. Bars represent mean ± s.d., n = 3. Values are normalized by GAPDH and compared with human H9 ESCs. (**E**) qRT-PCR for endogenous epithelial gene expression on reprogramming day 14. Bars represent mean ± s.d., n = 3. Values are normalized by GAPDH and compared with human H9 ESCs. (**F**) qRT-PCR for endogenous mesenchymal gene expression on reprogramming day 14. Bars represent mean ± s.d., n = 3. Values are normalized by GAPDH and compared with human H9 ESCs.

To understand how essential the stimulation of FOXH1 expression by NL + iDOT1L condition contributes to the enhanced reprogramming efficiency, we knocked  down FOXH1 expression using a specific retroviral shRNA construct described previously^7^. Inhibiting FOXH1 halted the formation of iPSC colonies by NL + iDOT1L condition (Fig. 3A), and virtually blocked the enhanced generation of total TRA-1-60+ colonies on reprogramming days 12 and 18 (Fig. 3B). The AP-stain positive colonies were similarly reduced (Fig. 3C). Surprisingly, we found in day 14 reprogrammed cells the endogenous core pluripotent gene expression except for NANOG was not obviously affected (Fig. 3D). Also, the epithelial marker genes were not changed (Fig. 3E). However, inhibiting FOXH1 expression resulted in significant (~6-fold) increase of SNAI2 expression, along with a general increase (though not statistic significant) of other mesenchymal markers compared with the NL + iDOT1L condition (Fig. 3F). These results indicate that the stimulation of FOXH1 constitutes a critical step for enhanced reprogramming efficiency by NL + iDOT1L condition, at least partially by suppressing key mesenchymal markers expression in reprogramming.

To investigate if FOXH1 is critical for OSKM + NL-mediated reprogramming in other cell types, we reprogrammed human dermal fibroblasts with OSKM + NL and observed massive formation of TRA-1-60+ colonies (more than two thousand TRA-1-60 positive colonies from 8.33 × 10^4^ starting cells in one well of 6-well plate). The blockage of FOXH1 led to a 2-fold reduction in TRA-1-60+ colonies (Figs. S1 and S2), which confirms that FOXH1 is also the downstream factor of NL enhanced reprogramming in fibroblast cells.

**Figure 4:**
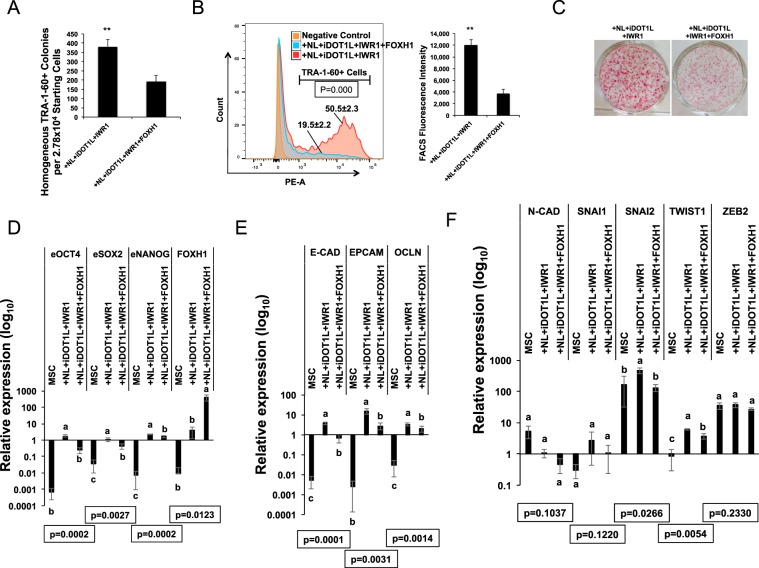
FOXH1 overexpression hindered NL and iDOT1L mediated reprogramming. (**A**) Numbers of TRA-1-60 homogeneously positive colonies induced in NL + iDOT1L + IWR1 control and FOXH1 overexpression conditions on reprogramming day 12. Bars represent mean ± s.d., n = 3. **p < 0.01. (**B**) (Left) Flow cytometry analysis of TRA-1-60 immunofluorescence in NL + iDOT1L + IWR1 control and FOXH1 overexpression conditions on reprogramming day 12. Percentage of TRA-1-60+ cell population in each condition is shown as mean ± s.d., n = 3. (Right) Mean fluorescence intensity of TRA-1-60+ cells in NL + iDOT1L + IWR1 control and FOXH1 overexpression conditions on reprogramming day 12. Bars represent mean ± s.d., n = 3. **p < 0.01. (**C**) Representative images for AP-staining of induced colonies in NL + iDOT1L + IWR1 control and FOXH1 overexpression conditions on reprogramming day 21. (**D**) qRT-PCR for endogenous core pluripotent genes and endogenous plus transgene FOXH1 expression on reprogramming day 14. Bars represent mean ± s.d., n = 3. Values are normalized by GAPDH and compared with human H9 ESCs. (**E**) qRT-PCR for endogenous epithelial genes on reprogramming day 14. Bars represent mean ± s.d., n = 3. Values are normalized by GAPDH and compared with human H9 ESCs. (**F**) qRT-PCR for endogenous mesenchymal genes on reprogramming day 14. Bars represent mean ± s.d., n = 3. Values are normalized by GAPDH and compared with human H9 ESCs.

By knocking down FOXH1 expression with another FOXH1 shRNA sequence reported previously^7^ (FOXH1-sh3), we further confirmed that FOXH1 is also necessary in OSKM + NL + iDOT1L + IWR1 reprogramming condition (Figs. S3, S4). No significant difference in cell proliferation or cell death was observed on reprogramming day 7 or day 9 by FOXH1 knock-downs (Figs. S5, S6). We then asked if FOXH1 overexpression would further enhance the reprogramming by OSKM + NL + iDOT1L + IWR1 condition, in which we found strikingly improved AP-stained colony number (Fig. 2C) and TRA-1-60+ cell population in reprogramming^19^. However, unlike the beneficial effect we observed in OSKM-mediated reprogramming (Fig. 1A, B), overexpressing FOXH1 caused ~50% reduction of TRA-1-60+ colonies in NL + iDOT1L + IWR1 condition on reprogramming day 12 (Fig. 4A). Fluorescence assisted cell-sorting (FACS) further revealed that FOXH1 overexpression caused dramatic reduction of TRA-1-60+ cell population among total reprogrammed cells on reprogramming day 14 (Fig. 4B, left panel). This is accompanied with significantly reduced TRA-1-60 expression level in TRA-1-60+ cell population (Fig. 4B, right panel). These results also correlated with an obviously reduced AP-staining intensity of the colonies induced by FOXH1 overexpression (Fig. 4C). These data indicated an inhibition of reprogramming process for NL + iDOT1L + IWR1 condition. qRT-PCR analysis of day 14 reprogrammed cells confirmed this by showing significant reduction of endogenous pluripotent gene expression including OCT4, SOX2, and NANOG with FOXH1 overexpression (Fig. 4D). Overexpressing FOXH1 in reprogramming also significantly inhibited the expression of epithelial markers E-CAD, EPCAM, and OCLN compared with the NL + iDOT1L + IWR1 condition (Fig. 4E). Also, consistent with our previous observation, FOXH1 overexpression significantly reduced the mesenchymal marker genes SNAI2 and TWIST1 compared with NL + iDOT1L + IWR1 condition (Fig. 4F). Thus, although endogenous FOXH1 expression is necessary for the NL + iDOT1L + IWR1 stimulated reprogramming efficiency, ectopic overexpression of FOXH1 thwarts further pluripotency establishment in reprogrammed cells.

## Discussion

Little is known about the upstream regulation of FOXH1, the ACTIVIN/NODAL signaling effector important for embryonic primitive streak development as well as for reprogramming. We found that in human cell reprogramming, NANOG and LIN28 significantly co-stimulate FOXH1 expression. Inhibiting DOT1L but not WNT activity synergistically stimulates FOXH1 expression with NL. We further show that blocking FOXH1 expression virtually diminishes the NL + iDOT1L enhanced reprogramming, indicating the essentialness of this gene expression in promoting reprogramming efficiency. However, although overexpressing FOXH1 mildly enhances the OSKM-mediated reprogramming, we found FOXH1 overexpression inhibits the pluripotency marker establishment in NL + iDOT1L + IWR1 reprogramming condition, thus preventing the maturation of reprogrammed cells. Our data demonstrate a proper FOXH1 expression level in reprogramming stimulated by NL and iDOT1L is needed for enhanced reprogramming.

LIN28 inhibits Let-7 miRNA maturation to promote the expression of Let-7 targets HMGA2, KRAS, MYC^22^, and HRAS in cancer cells^23,24^. However, other than MYC^1,2^, ectopic expression of HMGA2, KRAS, or HRAS failed to improve iPSC generation in human cell reprogramming^25^. We show here that LIN28 expression enhances FOXH1 expression in reprogramming. Recently, FOXH1 was identified as a target gene of Let-7 in Zebrafish^26^. It would be of great interest to investigate if FOXH1 is a Let-7 target in mammalian cells. Also, we show that NANOG stimulates FOXH1 in reprogramming. Whether this is through inhibition of Let-7 or through direct control of FOXH1 expression warrants further investigation. Furthermore, while endogenous FOXH1 is needed for NL + iDOT1L + IWR1 stimulated reprogramming, how overexpression of FOXH1 negatively interferes with complete reprogramming in this condition is worthy of future investigation. Overall our study elucidated a mechanism by LIN28 and NANOG to properly regulate FOXH1 expression as an important downstream effector for enhanced human cell reprogramming.

## Materials and Methods

### Chemicals and DNA constructs

The DOT1L inhibitor EPZ004777 was purchased from AOBIOUS Inc (Gloucester, MA, USA). Wnt inhibitor IWR1 was purchased from Selleckchem.com (Houston, TX, USA). The constructs pMXs-OCT4, NANOG, LIN28A, GLIS1, and FOXH1 were purchased from Addgene (Cambridge, MA, USA). Construction of the polycistronic vector pMXs-KMS was described in our previous study^27^. Retroviral shRNA construct specific for FOXH1^7^ were purchased from Addgene.

### Retrovirus packaging with 293 T cells

293 T cells were plated onto six-well plates at 2.5 × 10^6^ cells/plate. The next day, retroviral constructs, PUMVC and pCMV-VSVG (Addgene) plasmids were co-transfected into 293 T cell using Fugene 6 reagent (Promega, Madison, WI, USA). Cell culture media containing retrovirus were harvested at 48 and 72 hours post-transfection and filtered through a 0.8 *μ* m filter. The viruses were stored in −70 °C before use.

### Human somatic cell reprogramming

Primary human umbilical cord-derived mesenchymal stem cells (MSCs) from ATCC (Manassas, VA, USA) were maintained with low serum mesenchymal stem cell growth kit (ATCC). For reprogramming, on day −1, MSCs were plated onto six-well tissue culture plates at a density of 5 × 10^5^ cells/plate. On day 0, retrovirus carrying OSKM and other reprogramming factors were added with 10 μg/ml polybrene. The infected cells on day 4 were passaged onto mitomycin C treated mouse embryonic fibroblast (MEF) feeders in the presence of 10 μM Y-27632 (Selleckchem) ROCK inhibitor. On day 5, the medium was changed to a 1:1 mix of MSC medium and human ESC medium. Starting from day 7, the cells were maintained in complete human ESC medium, which contains 20% knockout serum replacement (KSR) in DMEM/F12, supplemented with 1 × NEAA, 1 × Glutamax, 0.5 × penicillin and streptomycin, 4 ng/ml human FGF2 (all from Thermo Fisher Scientific, Waltham, MA, USA), and 1 × β-mercaptoethanol (Merck Millipore, Billierica, MA, USA). iDOT1L and IWR1 were added in reprogramming as specified in the main text and maintained thereafter. The reprogramming of human dermal fibroblasts (ATCC) follows similar method and timeline except that fibroblasts were grown in medium containing DMEM plus 10% fetal bovine serum for the first 7 days of reprogramming before switching to human ESC medium without small chemicals.

### TRA-1-60 live staining and FACS analysis

For TRA-1-60 live-staining, the reprogrammed cells were stained with GloLIVE TRA-1-60 live stain antibodies (R&D Systems) based on the manufacturer’s protocol. Briefly, cells were incubated in reprogramming media containing TRA-1-60 antibodies at 1:100 dilution for 30 min, washed with DPBS and then continued to be cultured in reprogramming media. The stained colonies were visualized under a Nikon fluorescence microscope, with TRA-1-60+ colony numbers counted. For FACS analysis, cells were treated with TrypLE and resuspended in reprogramming media. Stained cells were then analyzed with a BD FACSCalibur flow cytometer with fluorescence excitation at 488 nm (BD Biosciences, San Jose, CA). FlowJo software was used for data analysis.

### Quantitative reverse transcription - PCR (qRT-PCR) analysis

Total RNAs were isolated from parental MSCs, reprogrammed MSCs, or human H9 ESCs with RNeasy mini kits (Qiagen, Hilden, Germany). Genomic DNAs were removed by DNase I (Qiagen) incubation. 0.5 μg RNAs were then reverse transcribed into cDNA using iScript reverse transcription supermix (Bio-Rad Laboratories, Hercules, CA, USA). qRT-PCR reactions were performed with SYBR Green supermix (Bimake, Houston, TX, USA) using the ABI 7500 Fast platform (Thermo Fisher Scientific). GAPDH was used as the housekeeping gene for gene expression normalization. Data were processed with the software associated with ABI 7500.

### Statistical analysis

Unless specifically indicated, all experiments were performed at least 3 times and data were shown as mean ± standard deviations (*s.d*.) of the mean. Statistical analysis was carried out using either ANOVA with Randomized Complete Block design (RCB) and LSD post hoc test with SAS 9.4. software, or two sample t-test with Minitab 18 platform. p value < 0.05 was considered to be significant.

## Supplementary information

Supplementary information

## Data Availability

All data generated or analyzed during this study are included in this published article, or are available from the corresponding author upon request.
