# Supplementary material for: FOXH1 Is Regulated by NANOG and LIN28 for Early-stage Reprogramming
Source: Sci Rep. 2019 Nov 11;9:16443. doi: 10.1038/s41598-019-52861-8 (PMC6848184; doi:10.1038/s41598-019-52861-8)
Supplement: Supplementary file 1 — Supplementary information [file 41598_2019_52861_MOESM1_ESM.pdf]

Supplementary information

FOXH1 Is Regulated by NANOG and LIN28 for Early-stage Reprogramming

Ling Wang, Yue Su, Chang Huang, Yexuan Yin, Jiaqi Zhu, Alec Knupp, Alexander Chu, Young Tang*

Department of Animal Science, Institute for Systems Genomics, University of Connecticut, 1390 Storrs Rd, Storrs, CT 06269, USA.

*** Corresponding Author:** Young Tang, Ph.D., Department of Animal Science, Institute for Systems Genomics, University of Connecticut, Storrs, CT, USA. Tel: +1-860-486-6619. Fax: +1-860-486-4375. Email: yong.tang@uconn.edu

Supplementary Figures

S1

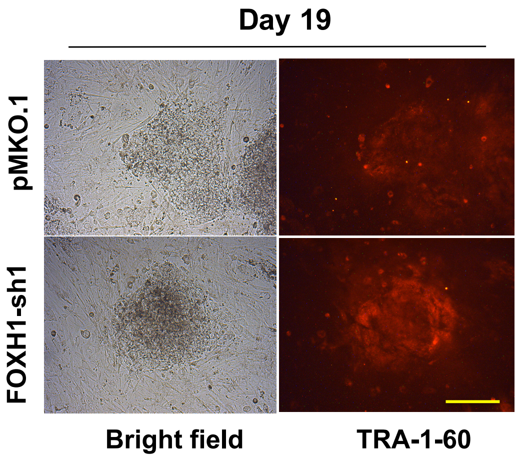

S2

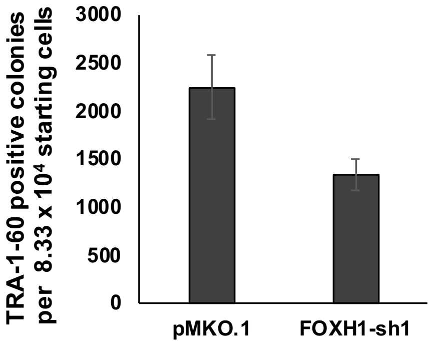

Figs. S1-S2 FOXH1 is also necessary for OSKMNL mediated reprogramming in human dermal fibroblasts.

S1 TRA-1-60 immunofluorescence on induced colonies from human dermal fibroblasts on reprogramming day 19. Scale bar: 250 µm.

S2 FOXH1 depletion resulted in a two-fold reduction in TRA-1-60 positive colony number. Bars represent mean ± s.d., n = 2.

S3

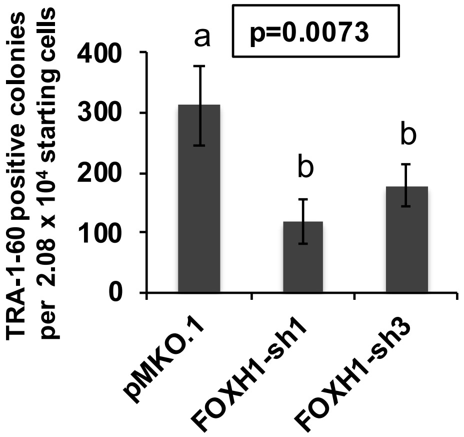

S4

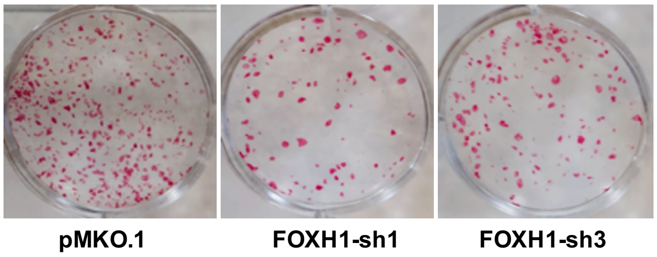

S5

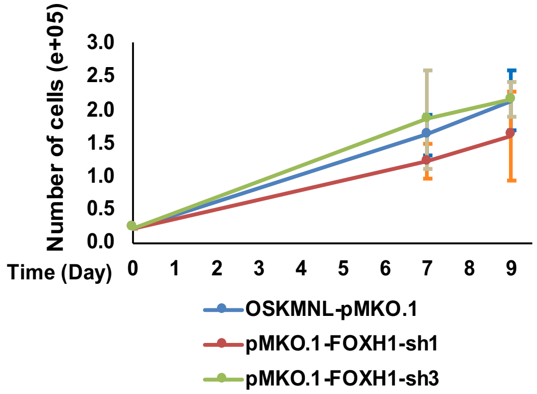

S6

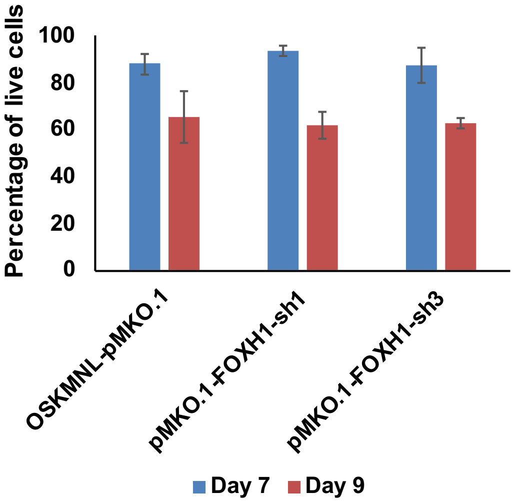

Figs. S3-S6 Depletion of FOXH1 inhibits reprogramming in OSKMNL+iDOT1L+IWR1 condition.

S3 TRA-1-60 positive colony numbers on reprogramming day 15 in human mesenchymal stem cells. Bars represent mean ± s.d., n = 4.

S4 AP stain of reprogrammed colonies on reprogramming day 21.

S5 FOXH1 depletion does not affect cell proliferation depletion during reprogramming. Bars represent mean ± s.d., n = 4.

S6 FOXH1 depletion does not affect cell viability during reprogramming. Bars represent mean ± s.d., n = 4.
